# Elements of the Pathology of the Human Mind

**Published:** 1839-04-01

**Authors:** 


### INSANITY.

I. ELEMENTS OF THE PATHOLOGY OF THE HUMAN MIND. By *Thomas Mayo*, M.D. F.R.S. &c.

II. TRAITÉ DES MALADIES MENTALES. Par *E. Esquirol*, M.D.

INSANITY is a subject on which our knowledge is progressive, and on which it requires to be so. An acquaintance with it implies an advanced state of pathological, as well as of mental science. It may be questioned whether either *is* advanced sufficiently to enable us to generalize very boldly, or conclude very certainly, on the nature and the laws of insanity.

But whatever may be the amount of knowledge or of ignorance on the part of those who have made insanity their study, it cannot be concealed that the bulk of the profession are far from well informed upon the subject. If proof of this were wanting, it would be found without difficulty in our courts of law, where the subtle advocate too frequently confounds the hesitating doctor. If one object of journalism is to augment knowledge, another, as important and more easy, is to diffuse it. We shall endeavour to do this in the present case, and, in as many articles as may seem required, to lay before our readers the last and the best investigations into the character and treatment of this frightful malady.

The object of Dr. Mayo would seem, from his preface, to be the exposition of his views on "moral" madness. A difficult part of a difficult subject. It is *in* the profession that some definite understanding must be come to in regard to it. The pleader, the judge, and the jury must ultimately take their tone from us. But at present we have no fixed opinions of our own, and our uncertainty and discrepancies furnish the legal armoury of logic and of dogmatism wielded against us.

Dr. Mayo's views may perhaps be guessed from the following passage in his Introductory Remarks.

"The medical art is not, as formerly, limited to the cure of specific and definite disease; its application is extended widely over our habitual and ordinary state. This fact is attested by the various works on diet, on dyspepsia, on the hygiene, which flow profusely from the press. But it should be remembered, that all that class of physical measures, which such considerations suggest, is calculated to produce a corresponding portion of mischief, unless the concomitant phenomena of mind are duly appreciated. A familiar illustration of this fact may be supplied from the disorders of the dyspeptic class of mankind. The dinner-pill, the careful choice of the most appropriate condiment, and the autumnal recreation of the stomach at Carlsbad, might easily be made to supersede various moral considerations, in smoothing the temper, obviating or preventing regrets, and counterbalancing disappointment. But, when it is considered, that a heavy reckoning awaits those, who rely on such palliatives as an adequate substitute for self-control, the admission, that an inquiry into the methods of obtaining this habit, should proceed *pari passu* with the direct physical expedient, will become obvious." 3.

The plan of his work is shortly told.

" A morbid state of mind may be said to exist, either where some property essential to mind in its normal state is perverted; or where some such property is abolished, or has been congenitally deficient.

The first class of those affections, in which these characteristics are found, I shall consider under the term insanity or madness.

The second class, I shall consider under two heads. That of brutality, or absence of the moral faculty; and that of imbecility or intellectual deficiency." 7.

Dr. Mayo wisely declines offering a definition of insanity. It does not admit of one, for insanity is a negative quantity, and the positive of which it is a negation is indefinite. When any one will define sanity, then we will engage to define insanity. Our author having refused to give a definition, proceeds to offer a metaphysical sketch of the phenomena of insanity, which ends, after all, in something very like a definition. For he winds up by remarking:—In regard then to the question, What is insanity? I answer that it is a morbid state, to which those persons are subject, in whom the power of volition is feeble, when they are placed under the influence of certain mental and physical causes. We think it will be admitted that this reply does not greatly elucidate our general idea of the complaint. As we feel averse, just at present, to enter on a metaphysical inquiry, we shall not examine either the proposition or the case on which it is founded.

In his second chapter, Dr. Mayo takes up the consideration of the moral and intellectual properties of the mind.

Following Mr. Dugald Stewart, he classes the moral or emotive properties under two heads:—such as lead to action, and imply an exertion of will, and such as are capable, indeed, of influencing the will, but do not *imply* any exertion of it. The first are active principles, the second are states and conditions of the mind.

" The above distinction is important to my present views, if I am correct in supposing, that the moral causes and preventives of insanity are to be looked for among the passive conditions of the mind.

Thus it is not to the love of power or of riches or of praise that insanity can be directly traced, when it springs up in the course of these several pursuits; it is to the regretfulness, the despondency, the timidity, the anxiousness, some one of which qualities, or of the others above noticed, will be found in the character in which the disease breaks out. The active principles, which may possibly be in play at the time, give occasion to the influence of these passive states; but they are not themselves causative of insanity." 20.

It does not appear to us that this hypothesis meets the case of madness from the gratification of desires. We believe there can be no doubt that a piece of good news has made a man insane. Joy as well as grief has unsettled reason, although triste emotions are infinitely the most powerful. Dr. Mayo makes some good observations on "regretfulness," and on "deficiency of hopefulness," and on "fearfulness." He passes to the moral faculty. We present the following observations on *it* and on selfishness, because we think them extremely correct.

"I mean, then, to express by selfishness that state of mind, which occasions us to find our enjoyment in any given line of conduct as serviceable to ourselves, and not as serviceable to others. Now this is a state highly predisponent to insanity, since it gives ample food to a noxious principle, which we have already noticed, namely, the tendency to regret and disappointment;—and that, on principles, which will readily be admitted. The wishes of a selfish man, to be a source of gratification to him, require the achievement of some definite object; and this, of course, he cannot always secure. The wishes of the benevolent, on the other hand, are accomplished, sufficiently at least for a large amount of gratification, if he can satisfy his mind, that he has done his best. But it is unnecessary to enter in to any proof of the fact, that our endeavours are more within our power than their success can be. Thus the stoics placed happiness in the possession of their good and wise man, because they supposed him to be concerned only with his pursuits and avoidances, and not with any definite acquisition; justly appreciating the fact, that happiness should be placed out of the reach of fortune, and that nothing is placed out of the reach of fortune except our endeavours.

I have next to consider the relation of the *moral faculty* to the subject of madness. When of sufficient strength to predominate over conduct, it is indeed a powerful antagonist, as lessening the number of our regrets. And this point it effects, not merely by the preference which it may be supposed to produce of right conduct over wrong; but also by contributing to the formation of a general principle, which is very rarely laid hold of by the mind, the principle of acting or abstaining from action because it is right. Men do not readily feel the pangs of regret, when they can refer the point of conduct to *any* settled rule. Dirk Hatterick's regrets were tranquillised by the reflection, that he had adhered to *his* one principle, namely, the keeping faith with his ship-owners. How much greater must their freedom be from regretful emotions, when they find their conduct in unison with a principle, with which the sympathies of human nature are in strict harmony." 31.

Dr. Mayo dwells strongly on the want of sympathy, as an evidence of an insane disposition or state. That the insanely predisposed are peculiarly deficient in this principle, or that insanity tends to weaken it, he thinks probable from the following considerations:—

First, that in the insane there is invariably a diminished state of those natural affections, which require sympathy for their full development, and these are the kindly ones. While, on the other hand, most persons retain their full capacity for the evolution of hatred, anger, and uncharitableness, properties which require no assistance from sympathy.

Secondly, that the supposition affords a practical diagnostic in insanity. In many persons thus situated, in whom, at the time at which the inquiry is made, the reasoning powers are in a clear and sound state, and whose emotions are at the time flowing evenly and quietly, we are enabled to detect disease, by observing, that the patient is not going along, or sympathising with us, or, indeed, with any one extrinsic to himself. Perhaps his conversation can only be characterised as morbid, by its remarkable want of relation to all that is passing in the minds of by-standers. In this point, indeed, the insane are remarkably contrasted with the inebriated, whom, in many respects, they greatly resemble. Persons in the latter state overflow with sympathy, and carry it to a ridiculous and maudlin extent.

Dr. Mayo's theory of the production of insanity would tend to resolve the complaint very nearly into the predominance of triste emotions. It may admit of question whether he has not pushed his hypothesis rather far, but, in the main, we are disposed to think that he is right.

He concludes the chapter by the following brief allusion to "double consciousness."

"Among the more obscure and dubious topics, out of which light may at some future time be shed upon this mysterious subject, the following perhaps deserves a place.

There is a morbid state of the human mind admitted by pathologists, under which the patient lives in alternate stages, as it were of two different beings, in regard to the sequence of his thoughts, and the operations of his intellectual and moral properties. The one is easily recognised as his normal state. It exhibits the ordinary phenomena of his character and habits. In the other he appears to have undergone a remarkable change. He has forgotten things and persons, or views them in perfectly new lights. The current of his thoughts verges on delirium in rapidity and excitation. Sometimes there appears in him more force and vivacity of intellect in his paroxysmal state, than was observable in his original character. From each of these states he drops suddenly into the other, and he has no clear recollection in the one, of subjects which had interested him in the other. This morbid state, to which the name double consciousness is usually given, has a considerable affinity to the intermittent form of madness; so much so, that it seems not unreasonable to suspect that their laws of causation may have some common points." 38.

An allusion to Mesmerism, which succeeds, is, we think, so far from happy, that we shall not quote it. No amount of merit will be able to support the burthen of the slightest credence in that compound of folly and imposture.

We pass on to an examination of the intellectual properties. These, like the moral feelings, may be viewed as active principles, or passive states.

The active intellectual principles are undoubtedly consistent with mental health, and, rightly directed, are preventive of mental disease. As Esquirol has remarked, the predisposed to insanity require a special education; and were this understood and resorted to, many a case of lunacy would be prevented. Nay, as Dr. Mayo justly observes, the faculty of observation may be made available towards even the removal of actual insanity. No one, who has witnessed the temporary mitigation of insanity produced by the studies, to which this faculty leads, can doubt its efficacy. A flower, an insect, or a mineral, have often beguiled the restlessness of that disease into temporary self-control, when the mind had been previously imbued with a taste for such pursuits.

We agree, too, with Dr. Mayo in the following assertion of the delightful character of literary labour.

"The same remark applies to the active operations of the intellect, which we have formerly made, in regard to the benevolent affections; and it has an equally close connection with our present subject. *They* do not depend for their gratification upon success in the pursuit of their objects. Their labours are their reward. Probably the happiest men in existence, 'sua si bona norint,' are persons engaged in literature and science, as an employment. It is to be regretted, that they are not always sufficiently aware of the above remunerative points in the nature of their exertions. The reason of this is probably to be found in the extensive tendency of those who conduct the education of our youth to encourage ambition as the principle motive to such exertions." 42.

Probably, the happiest man in existence is he who combines with intellectual tastes and occupations a well regulated morale. The former indeed *tend* strongly to confirm the latter, for intellectual appetites generate knowledge, and knowledge is father to virtue. The pursuits of ambition and of pleasure carry in their stimulus the seeds of exhaustion and disgust. Literary tastes last as long as the mind itself, and please while the faculties admit of pleasure from the objects around us. Contrast the days of Sir William Temple or Sir William Jones with those of Fox or Pitt. As regards the happiness of the *individuals*, how far the two first went beyond the party statesmen !

Dr Mayo proceeds :—

"There may be, and in some cases I have witnessed, a very beneficial use of intellectual power, in averting insanity, when the patient has been able to apply the resources of his understanding to the regulation of his mind, under a consciousness of his own predisposition. It is needless to observe, that the possession of very high talents is presupposed in him, who can be entrusted with such an application of his intellect. But insanity constitutes no exception to the great law of our nature, which in some degree places in our own hands the remedies of all the diseases with which we are visited." 43.

He quotes with approbation and delight from a beautiful letter of the late Sir James Mackintosh's, in which he ascribes the possibility of such self-surveillance to his unfortunate friend Robert Hall.

"'That the mind of a good man,' he observes, 'may approach independence of external things, is a truth which no man ever doubted who was worthy to understand it: but you, perhaps, afford the first example of the moral nature, looking at the understanding itself as something that is only the first of its instruments. I cannot think of this without a secret elevation of soul, not unattended, I hope, with improvement. You are, perhaps, the first that has reached this superiority. With so fine an understanding, you have the humility to consider its disturbance as a blessing, so far as it improves your moral system. The same principles, however, lead you to keep every instrument of duty and usefulness in repair, and the same habits of feeling will afford you the best chance of doing so.

We are all accustomed to contemplate with pleasure the suspension of the ordinary operations of the understanding in sleep, and to be even amused by its nightly wanderings from its course in dreams. From the commanding eminence which you have gained, you will gradually familiarize your mind to consider its other aberrations as only more rare than sleep and dreams ; and in process of time they will cease to appear to you much more horrible. You will then be delivered from that constant dread, which so often brings on the very evil dreaded; and which, as it clouds the whole of human life, is itself a greater calamity than any temporary disease. Some dread of this sort darkened the days of Johnson ; and the fears of Rousseau seem constantly to have realized themselves.—But, whoever has brought himself to consider a disease of the brain as differing only in degree from a disease of the lungs, has robbed it of that mysterious horror, which forms its chief malignity. If he were to do this by undervaluing intellect, he would indeed gain only a low quiet at the expense of mental dignity. But you do it by feeling the superiority of a moral nature over intellect itself.' " 45.

There is certainly something infinitely noble and ennobling in the preceding sentiments. And no doubt there is much truth in them. For although it is certain that the causes of insanity are too often physical and uncontrollable, it is equally certain that they are occasionally found in the operations of the understanding, rather than in the structure of its organ.

Dr. Mayo makes Imagination the subject of his Fourth Chapter. His object is to shew that, applied to intellectual operations it is a source of mental health—and, that, linked with the emotive, it is not. This Chapter, and the Fifth, on the Relation of Phrenology to the Science of Mind, we pass over.

But we pause at the Sixth, which is occupied with the Pathology of Insanity.

This is considered by Dr. Mayo under three stages.

In the first stage the deviation from soundness of mind regards moral conduct.

In the second stage the moral incoherency continues ; but intellectual incoherency or delirium has also taken place.

In the third stage, recovery from the above states is proceeding;—or the patient is gradually passing into a chronic state of moral and intellectual perversion.

"The prominent features of the first stage are a heightened condition of those moral defects or excesses, which I have noticed among the causes of insanity. The patient is regretful, he is despondent, he is suspicious, he is anxious on the subject of property, or personally fearful in an increased degree. His imagination is probably in an excited state. The points which distinguish such attributes in *him*, from the same *kind* of attribute in one to whom we should refuse the appellation of insanity, are as follows. The extent of his sufferings lose all their accustomed proportion to their causes. The surmises, from which he reasons and converses, are utterly incongruous with those of other men. Thus he is the object of an inconceivable conspiracy. He is noticed and remarked upon by persons, to whom he must be utterly indifferent. A woman of a pure and correct mind will suppose herself in love with some person, whom she has never seen or beloved and persecuted with addresses by such a person. She will be unwilling to go to church, because every thing that the preacher says, is meant to apply personally to *her*. The kindliness of her character is destroyed, and intense selfishness takes its place. Indeed the blunted state of natural affection is a remarkable trait of commencing insanity. The sequence of her actions becomes incongruous and inconsistent. Among all these peculiarities the powerless state of the insane *will* is distinctly traceable. This diagnostic is important in its relation to another disease, with which insanity may be easily confounded ; namely, hysteriæ. It suggests indeed this difference of practice, that the hysterical should be treated as far as is consistent with humanity, as if they could avoid or avert their morbid state ; a procedure, which would utterly fail if applied to the insane." 63.

It would be difficult to draw the line between this stage of insanity and hypochondriasis. In fact the deviation from what we term sanity up to what is palpably insanity, is so gradual and progressive, that it would be impossible to determine the limits of the several transition states. Take so decided a case as the one Dr. Mayo has painted, and no one can reasonably doubt its nature. But there are many instances of a more indefinite character, where we feel inclined to pronounce a man insane, yet would experience no little difficulty were we to attempt to prove him so. These are the cases which are frequently occurring, ruining the peace and blighting the prospects of families, and often giving rise to protracted and costly litigation. It would be well had we any criterion of this stage of insanity. Unfortunately we have no absolute rule by which we may determine its existence.

Dr. Mayo proceeds to shew, or to endeavour to shew, that this stage of insanity takes its tone from the temperament of the individual—that the bilious exhibit oppression—the leuco-phlegmatic, languor—the nervous, tremor—the sanguineous, fulness. Yet insanity is essentially a debilitating state, and throughout the temperaments, there is a dry and unwholesome state of the skin.

The second stage is next considered by our author.

Inconsequential conduct, he observes, has probably continued for some time, the duration varying in different cases; and now the state of inconsequential thought presents itself. The erroneous impressions, which have influenced conduct in the first stage, and which even then might perhaps have been traced to false perceptions, are now palpably founded on such impressions. As the patient happens to be more sanguine or more nervous in his temperament, so much the more sudden and acute is this change. It is slower, and more gradual in the bilious, and most so in the leucophlegmatic.

This state is delirium, such at least appears to be the sense, in which that term is ordinarily used; namely, as involving inconsequential thought, and false perceptions.

Dr. Mayo endeavours to point out the distinction between the delirium of phrenitis and of insanity. In the delirium of phrenitis and of fever, he says, so far as any definite ideas or propositions are formed in the mind, they are inconsequential, and so far partake of the character of insanity. But under the ravings of phrenitis, propositions are rarely constructed. The delirium of insanity disturbs the sequence of ideas, and renders them unsuitable to external objects; but leaves the ideas definite, though erroneous and inconsecutive. Thus the discourse of an insane person will bear to be divided into propositions; that of a phrenitic patient will not. It is a process of ejaculations. Words tumble out casually, and take no logical relation to each other.

The sensations of the insane are rarely acute. They easily pass into a state of exhaustion and of sinking—a fact of great importance in reference to treatment.

The third stage may be said to differ from the second at its commencement in degree rather than in kind. The power of regulating the trains of thought is equally wanting. The falseness of perceptions continues, but the current of thought is flowing less rapidly, and where the case tends towards recovery the patient can be induced to doubt the justness of his perceptions. It is the same with the physical symptoms. Both they and the mental ones experience different modifications afterwards, according to the extent of the disease, and the patient's capacity for recovery. In many the state of quiet desolation with which this stage commences, is permanent. The unhappy patients are irrecoverable. In many again, this state subsides into what is called a lucid interval. The patient is at first supposed to have recovered. But at the end of a term of weeks, he has a repetition of the second stage; and the disorder proceeds upon this principle of alternation. This is an unfavourable form of the complaint, but not an incurable one. In the more fortunate instances, the powers of self-control are gradually resumed, and the mind regains its normal integrity.

In some instances, the moral symptoms, which our author supposes to form the mental indications of the first stage, are so indistinct as to escape attention. The disease has arrived at its stage of development before it is actually recognized—an argument in Dr. Mayo's opinion, for carefully scrutinizing and rightly understanding the premonitory moral symptoms.

The Seventh Chapter examines the connexion of Suicide with Insanity. We think that the observations we shall quote contain much truth.

"That the tendency to suicide should be an occasional concomitant to insanity might be expected; since the sufferings, which produce this mental disease, also render the possession of life less desirable. It is also true, that insanity facilitates the commission of suicide, by removing in some degree and in some cases the barrier opposed to it by the dislike of bodily pain; since, with diminished susceptibility of bodily pain, the fear of inflicting it upon ourselves must also be diminished. When, however, certain elements of character, such as a quick sense, and an impatience of moral suffering are present in a high degree, or when, existing actually in a lower degree, they are excited and fostered by indulgence; when again these qualities co-exist with a physical indifference to pain and danger, they certainly predispose to suicide, even where no insanity may be present. If external sources of unhappiness are added, or, if physical constitution, such as some forms of bilious indigestion, co-operate, the risk is increased.

Let us suppose a given individual predisposed to suicide by a concurrence of these causes; and let us suppose, that insanity has supervened. It will be observed that the danger of suicide is augmented in the supposed case, or, on the other hand, is occasionally diminished, on grounds, which it is important to trace.

When insanity has proceeded to the extent of obliterating all consecutive thought, the patient is placed under less risk of suicide than he was under before. When, again, the state of insanity is less complete, so that it leaves the operations of thought still energetic, and only destroys our power of self-control,—insanity must then invigorate the tendency to self-destruction, and increase the risk. On the whole, the cases saved from suicide by the obliteration of thought will not be sufficiently numerous to prevent the very frequent co-existence of this act with the mental disease.

This method of viewing the subject of suicide has always appeared to me more philosophical, than the regarding an act so deliberately performed, as it often is, and with so tangible a motive, as involving necessary insanity." 72.

Dr. Mayo goes on to observe that, without the assistance of revealed religion, reason alone has never been able to make out a strong case against the commission of suicide. If unquestionable evils are irremediable, reason can offer no valid argument why they should not be escaped by voluntary death. It is very well to urge that this is cowardice. So it is; but the avoidance of all evils is cowardice in this sense. It is true, that the exhibition of such stoicism as enables a man to bear up against "a sea of troubles" is a noble one, more noble than the spectacle of his escape from them by self-destruction. Yet this is no conclusive argument—it is an appeal to the moral courage of the self-destroyer, an appeal which he may meet by the counter-affirmation, which every man's feelings tell him has *some* force—" You say that I shew a want of moral courage in flying from misery to voluntary death. But is not self-destruction, that 'leap in the dark,' an act of some degree of courage too—a victory over the strongest of our instincts, as well as over our sense of the reprobation of mankind?"

As an argument, it is difficult to parry this defence of suicide. Fortunately revealed Religion becomes the arbiter, and authoritatively denounces as a sin against God, what Reason finds it hard to demonstrate a crime against herself.

If it be true that the inconsistency of suicide with reason is incapable of proof, it follows that suicide does not necessarily imply insanity. For revealed religion will not influence unbelievers nor those who may never have been taught its blessings. Nay, Christians may believe, and yet incur the guilt of suicide, just as they may perpetrate another *crime;* the incentives to it overpowering the sense of the Divine injunction.

It has always seemed to us that the doctrine which affirms suicide to be itself a proof of insanity, is weak in argument, however beneficial in its consequences. It reasons in a circle, because it makes the terms of the proposition their own proof: " suicide implies insanity, because a man who conquers the instinct of self-preservation is insane." We have already observed that this is far from certain—in fact, the self-destroyer may reply with plausibility—" you who consider mere life the great good, and prefer existence branded with ignominy, or sordid with misery, under all circumstances, to self-inflicted death, have a mere animal horror of dying, and evince a want of the higher qualities of our moral nature, a keen sense of degradation and injustice."

It would not be easy to shew that the suicide of a detected criminal or a patriot persecuted to the last extremity, of a Fauntleroy, or of a Cato, is primâ facie evidence of loss of reason. All, in fact, hinges on this assumption—*that,* " the victory over the instinct of self-preservation is so contrary to nature as to imply the fall of reason." There is, alas! some force in the arguments which go to shew that such a victory is, occasionally, Reason's triumph—a step from the brute, not towards him.

If the principle that suicide implies insanity has become universal in practice, it is, we fancy, because experience has shewn the inutility of the contrary doctrine, while humanity has revolted against its barbarous consequences. The cross-road burials, the torches, and the stakes of a less enlightened age are too skocking for the refinement of the present. Suicide is a crime against God rather than man, and the unhappy perpetrator of the dreadful act should be left to the mercy of his Almighty Judge.

Dr. Mayo offers the following summary of considerations in reference to suicide:—

The suicidal tendency belongs principally to the bilious temperament.

It is rarely found in persons of personal timidity or of moral courage.

It is remarkable for the intensity with which it operates, when developed in the insane, and for the address with which it masques its measures.

Accordingly, it increases the difficulty of treatment, whenever it co-exists with the insane state; and nothing but the most watchful attention can defeat the manœuvres by which the unfortunate patient endeavours to attain his purpose.

"One of the first points," he continues, "which require consideration with this view, is the quality and kind of false perceptions occurring in a given case. Some lunatics will be found adopting their daily line of conduct, or again changing it, in reference to certain voices, which appear to dictate to them. Such persons are liable to carry into effect whatever measures are uppermost in their own mind, since these measures derive oracular force and influence from being conveyed, as it were, into their ears by their invisible companions. Through questions adroitly put, as to the communications thus made to him, the patient's own intentions may often be obtained and anticipated. And this leads me to notice a rule of practice extremely important in regard to the general interests of the patient, as well as to our present topic. In discussing with him his false perceptions, while we assert, that he stands alone, or, as was well expressed to me by an insane person, that he is in a 'minority of one,' in regard to them, we must be cautious, how we deny their reality, that is, their reality in regard to his perceptions, and much more how we deride him. By neglecting this rule, we often lose a valuable means of ascertaining the springs of action, by which he is influenced, and thus of defeating improper designs, and generally we diminish his confidence in our promises and assertions. For the lunatic is not likely to feel this confidence, if feelings which are important to him, are derided; and if phenomena, which present themselves to *his* senses, are ascribed to his imagination. Meanwhile, the existence of this confidence is perfectly compatible with a distinct assertion, and protest on the part of the physician or the friend, that *he* does not participate in those views which the patient describes as his own. When this relative reality is admitted, the sufferer is induced to accept the assurances with which they are accompanied, and to believe, that they may at some future time turn out equally correct in other points." 79.

Exuberant gaiety in one whose ordinary state is extreme depression is ominous. The patient has, perhaps, confirmed his resolution for self-destruction.

Dr. Mayo alludes, but too cursorily to render it necessary to follow him, to the homicidal tendency, occurring without an intelligible motive.

The Eighth Chapter is dedicated to the preventive treatment of insanity by mental means. Like M. Esquirol, our author dwells on the advantages of judicious early mental cultivation. But we pass to the period when the disease is actually commencing. Surveillance is necessary. The following are Dr. Mayo's precepts:—

"Do not scold your patient. He wants an opiate at your hands; and as the drug itself rarely agrees with him, you must give it him in a moral form. Endeavour to relax the tension of thought, and feeling. Help him into listlessness. Meanwhile, though you argue no point with him, remember, that you must concede no measure. The more resolved he finds you, provided your firmness is tempered with kindness, the more he will rely upon you. He needs your support against his own vagueness and instability.

Check your patient, and teach him to check himself, at moments at which he is endeavouring to secure too large a measure of positive enjoyment. The measure of enjoyment, which every man should consider appropriate to his case, is just as much as can last its time, without occasioning nervous excitement.

If your patient is regretful, let him accustom himself to assume that the recurrence of regretful feelings is a law of his constitution; and to consider the force of each re-action, as commensurate, not with its reasonableness, but with the strength of the impulse, under which he had previously acted or resolved to act." 96.

When actual delirium supervenes, separation becomes indispensable. Nor need we dwell on its advantages. With the third stage come, observes our author, the tempora fandi; the opportunities of an effectiver emark, must be observed with watchful attention. Suggestions vividly and pointedly made of incongruities of thought and conduct often appear to rouse the patient into a consciousness of his state. His mind perhaps darkens again, but the vestiges of an impression thus made are often observable in his subsequent conduct. Many such alternations of light and darkness occur during convalescence.

Dr. M. thinks that a valuable influence may be obtained from the operation of sympathy. It has occurred to him, in many instances, to observe the effect of a well-regulated mind upon the convalescent insane, when that mind is furnished with the curious tenacula of sympathy, which seize and hold under their influence the minds of others. Instances, however, have occurred, in which the influence has been reciprocal. Dr. M. relates a case in point. An anxious and attached wife had maintained a long-continued intercourse by letter and conversation with her insane husband, who resided at a private establishment. This gentleman had passed into the third stage of his complaint; and there continued. He required but little surveillance, and united in a remarkable degree his original capacity for clear thought and sagacious reflection with the rapid and erratic associations of insanity. The lady herself, though nervous by temperament, had a sound and strong understanding. In talking with her, however, it is easy to see, that the sequence of her thoughts, and the links by which they are connected, have been influenced by the habitual contemplation of a morbid mind. Their mental relation has indeed become that which is expressed by the terms "ils s'entendent."

"There is much skill," continues Dr. Mayo, "required in the management of the insane, in observing a distinction between those ideas, which belong to his disease, and those, in regard to which his mind is *at the time* not insane. The powei of control over the train of thought sometimes returns very suddenly: and it is of immense importance, that the chain thus recovered should not escape the patient's grasp. Now, whatever modes of thought receive the sanction of a judicious medical attendant, are by this circumstance in some degree recommended to the attention of the patient, provided his sympathies have been secured. Thus his recovery becomes valid in his own eyes, when countersigned, as it were, by the opinion of his friend.

A patient, who had been insane for three years, and had spent a large portion of that time at the establishment at Ticehurst, had passed into a state of alternate lucid intervals and paroxysms, each of these states successively lasting for some weeks. At the commencement of one of these, he announced to Mr. Newington, the proprietor of the establishment, that he should never have another attach. On learning this, as well as that he had never before made any similar remarks, I went over to Ticehurst; and formally stated to the patient, that I accepted with pleasure his announcement of his recovery; that I believed he was correct in his supposition that it had taken place; that nothing more remained, than that he should give himself and me some proof of the soundness of his own impression by spending a portion of time, which I named, at the establishment. This patient never relapsed. 103.

Dr. Mayo's sentiments on the physical treatment of insanity are judicious. They apply to the use of depletory agents—of tonics—of sedatives—and of counter-irritants. He joins in reprobating that extreme depletion, which, we fear, is too often practised in mania. He relates a striking instance of the possible bad effects of even a small loss of blood. It was the case of a middle-aged lady residing at an establishment, in whom the disease had continued for some time in its first stage; the application of a few leeches to the temples, which her dull and oppressed look and robust figure seemed to indicate, as, at least, a safe measure, gave a very mischievous development to the disease. She became instantly very delirious, laughing and chattering incessantly, and in this state, Dr. M. found her about four months afterwards. He relates another case in the patient's own words as a sample of one in which depletion would probably have been injurious.

A lady had, by Dr. M.'s direction, taken some mild alterative aperients, and a cordial mixture for six days. "I still feel," she says, "a whirl about my head. I should describe it as if it felt too tight; and express the feeling, as if air got in and made a whistling or rushing about my ears; *or I could fancy it voices talking to me.* I feel a reluctance to apply to anything; to work a sum with my children seems too great a strain on the forehead; and I forget as soon as I have read a page, or indeed any conversation; even, if I desire it to make an impression. I once, many years ago in 1818, had a slight illness in Paris, and possibly from over-excitement a tendency to imagine things different from what they really were. This always made me most anxious during my confinement, and at other times, to keep myself as tranquil as possible. I never had any return. But have for some time felt unwilling to attempt mental exertion. I remember, once at ——, taking some bark and cayenne pepper; and it seemed to clear my ideas and to make my mind more collected." The lady was a fine person, aged about thirty-five, of a full but flaccid figure, her temperament leucophlegmatic and nervous; but, living in a very bracing air, she kept herself in a state of fulness, which might easily simulate the robust sanguine constitution. She had had several children; and her mind was kept on a stretch by domestic cares. The catamenia were free in quantity and regular. Now in this case, the lancet, if used for the above congestive symptoms, would have had a very mischievous effect on her powers of self-control.

Moderate depletion is well borne by the melancholic. But free venesection may convert quiet melancholia into delirium.

In the case of a young lady, the accidental taking of a drachm of nitrate of potass by mistake for a scruple three times a day for a fortnight, which produced bloody evacuations and obstinate vomiting, appeared to operate most beneficially in the disease. It subsided with going into its second stage, and has never recurred.

Dr. Mayo considers tonics valuable in the first stage of insanity, in the serous and the nervous temperaments, mischievous in the bilious and sanguineous.

In the second stage tonics are inapplicable, whatever may be the temperament of the patient.

In the third stage, they are valuable in every temperament, with this reservation, that their use in each should be cautious or bold, in the same proportion, as it may be noxious or salutary in the first stage of the disorder under that temperament.

Of course Dr. M. speaks well of purgatives. And he concurs with the best practitioners in extolling sedatives.

"The intention of sedatives is in every stage of the disease a wise one. By soothing the insane patient, we at once give him wholesome strength, and reduce morbid action. Of all the medicines which possess this virtue, opium has been in my experience the least valuable, and digitalis the most so. The following recipe I have found very useful in the second stage of the sanguine or sanguineo-nervous temperament. Mixturæ camphoræ ℥xij.; potassæ nitrat. ℈j.; tincturæ digitalis ♏xv. M. fiat haust. ter quotidie sumend." 117.

Dr. M. speaks disparagingly of opium—commends the extracts of henbane and lettuce, combined with camphor in small doses, and with the compound extract of colocynth—lauds the infusion and tincture of hop—and leans dubiously to counter-irritation, particularly in the bilious, leuco-phlegmatic, and sanguine temperaments.

The Tenth Chapter is occupied with Brutality. This was the subject of a former essay by the same author. The name is retained—the thing is described as before—but the view in which it is regarded is altered; for he *did* look on it as a form of insanity, while he *does* regard it as destitution of principle. We must say that if brutality is to be deemed insanity, there is no crime so monstrous, no deviation from the path of natural feeling so wide, as not to claim the privilege of the same plea. Tiberius, Caracalla, would cease to disgust—they would become objects of pity. The executioner might burn his halter, our criminal courts might be turned into lunatic asylums, for the great criminals, the Thurtells and the Greenacres, would no longer be the victims of offended justice. Our penal code would be almost limited to petty larceny and shop-lifting, and with the progress of science, even these would be found at last to be only forms of madness. Take Dr. Mayo's description of brutality, and see if it be not what is usually deemed a vicious tendency unchecked by proper education, or ripened by indulgence.

"We have a class of persons, differing from the majority of mankind in their incapacity for moral distinctions, differing from the insane, in not labouring under any suspension of the power of will. On the first of these grounds they have a right to a place in our system of mental pathology. On the last, they must constitute a distinct head from insanity. I am not at present considering this class generally; I exclude indeed that section of persons, in whom the absence of principle is obviated by the harmlessness of their tendencies. I am speaking of persons destitute of the moral faculty, and *also* vicious in their propensities. For these I have borrowed the designation given to them by Aristotle; and I call them *brutal*.

In regard to the principles on which this morbid condition may be treated, the law, it may be observed, greedily takes advantage of its co-existence with insanity, whenever this occurs, and it readily does occur, to control the unsound habit of mind, but has not hitherto been able to grasp it in its own form. Although in truth, the state which we term brutality spreads as wide devastation as insanity would, if insanity were left uncontrolled; and is, according to the above view, equally a disease of mind." 128.

If it be difficult to determine the existence of insanity in its usual forms, how impossible it would be in practice to draw a line between "brutality" and vice or crime. The latter is punishable in a sane man, because the exercise of reason enables him to perceive that it is culpable, and that the laws of society denounce it. Has the " brute" this power of reflection and of estimation of consequences, or not? If he has, he is a responsible agent —if he has not, that can be proved by aberrations of reason.

Dr. Mayo seems to hint that the law might reach brutality. But if brutality be rational how can any law reach it? The law cannot *punish* tendencies. How are they to be determined? Only, we presume, by acts. But if acts be culpable they are already punishable by as strict a code as the circumstances of society permit. If they are not culpable, we do not see how the law can grapple with them. Law must define, must say what it will and what it will not suffer. If it did not say this, none would be safe; the powerful would escape—the weak would be hunted down. No doubt there are acts without the scope of law which are offences against our moral sense, and even injurious to society. But there are punishments, as well as crimes, independently of those with which law deals—acts which entail retribution in a thousand ways—which make the tyrannous father suffer from the disobedience of his child: the harsh husband from the indifference, the drunkenness, or the infidelity of his wife: the proud or the imperious man from the desertion of his friends. We are sure that it is impracticable for law to deal with anything but facts, or to regulate society and repress vicious tendencies, connate or acquired, by any other means than by the punishment of those acts which she denounces.

Dr. Mayo adds:—

"It is indeed time, that the disgraceful scenes should terminate, which are now occasionally enacted at the public offices in London; where a father is heard requesting that an ignominious punishment should be inflicted upon his son, as the only moral expedient which can reach him; and thus finds no other mode of obviating the deficiency of principle, than the *penal* inflictions of the law. But it is yet more painful, that the offender should be allowed to wander on through crimes and inflicted misery, until he reach this goal. An instance of such a termination to the course of the brutal person is afforded by the unhappy Lord Ferrers. That nobleman was not *insane* in any customary use of the word; his intellectual faculties were good; and they were directed by a powerful will towards definite objects; neither did he exhibit that moral incoherency, which we have described among the earliest phenomena of the insane state. The business-like talents, indeed, which he displayed in his own defence, indisposed his judges to allow him the advantage of *that* plea. But his brutality made him unfit for *social* existence: the laws of this country did not reach him as a subject for confinement. Therefore he was hanged. This procedure was unavoidable under the circumstances of the case, and in the present state of our laws; but it constitutes a painful fact, considering, that education at present affords no preventive to such criminality." 132.

We do not think that Dr. Mayo proves his case. Brutality, such as he describes it, does not seem to us to require, on the part of society, the shield of insanity against legal consequences, nor to merit it on the part of the individual. If " brutality" were allowed that defence, it must be extended to almost all the ramifications of violence, of passion indulged, and of evil propensities uncontrolled. The case to which Dr. Mayo refers—that of the father and son at the police-office—is frequent enough no doubt. But it shews no more than that the father has educated that son amiss, and that bad associates, and corrupt habits, have completed what parental mismanagement began.

Unless it can be shewn that vice is attended with deficiency or alteration of the reasoning faculty, it must, and, for the interests of mankind, it ought, to be held responsible for its mis-deeds. Education tends to prevent it. But moral education is a private affair. The state cannot take each child from the cradle, and regulate the nursery or parlour. That must be left to parent and preceptor. If they fail to pluck out the seeds of wrong doing, and allow them to germinate and ripen, woe to the unhappy being in whom they have been let grow, for his only safeguard against himself is his terror of the strong arm of insulted law. This, indeed, is admitted by Dr. Mayo, and, making that admission, he withdraws "brutality" from the pale of irresponsible mental infirmity, and places it, where it ought to be placed, within the catalogue of guilty things.

The Eleventh Chapter, on Idiocy, congenital or acquired, presents nothing of moment. It is more easy of appreciation than some other forms of insanity. The degree of deficiency of intellect which constitutes it can only be determined by special examination in each particular case.

The Twelfth Chapter is intended to supply some omissions in its predecessors. *They* cannot be considered by our author great, for *it* is extremely brief. The only passage that we think it necessary to introduce is the following:—

"In describing *medical* treatment I have to regret a very important omission; namely, the use of nauseating remedies in this disease. The large doses in which ipecacuanha and the tartrate of antimony are borne by the patient, without exciting the effort to vomit, and the decisive effect which they produce in shortening and mitigating the second stage of the disease, entitle them to the highest attention. In recommending measures that may tranquillise the insane *mind*, I have omitted the important topic of *employment*. This, indeed, if cautiously managed, conduces to tranquillity. Gentle manual employment is of great avail in diverting mental irritation: and in this point the habits of females give them a valuable advantage. Of the intellectual faculties, that of observation may be cultivated with the greatest advantage; it does not *imply* the continuity of action, which easily runs on into incoherent thought in the insane intellect; and it tends gradually to wean such an intellect from false perceptions." 152.

We take our leave of Dr. Mayo. His little work will repay perusal. It cannot be considered an elementary treatise, but it contains many judicious reflections, and the reader will probably rise from its perusal better qualified and more disposed to study the mental phenomena of insanity, and to regulate the insane.

### II. ON HALLUCINATIONS.

These form the subject of the second essay of M. Esquirol, and occupy forty-three of his pages.

There is a certain form of insanity in which the individual believes in the reality of impressions, sometimes transmitted by one sense, sometimes by another, at a time when no external object really exists to give rise to them. One person hears voices, questions, replies, keeps up a connected conversation, is insulted, threatened, has orders addressed to him—he disputes, grieves, or gets into a passion—is saluted with celestial harmony, with the warbling of birds, with a regular concert; but the sounds are all the while imaginary, and silence reigns around him.

Another gazes on the most varied scenes, sees the Almighty himself, sits at a play, greets the familiar countenance of a friend—or a precipice yawns before him, armed foes are there to assassinate him, serpents are hissing to devour him; and this individual is in darkness or in blindness.

A person in this state sees a car of light waiting to bear him to heaven; he opens his window, steps forth to enter it, and is precipitated, of course, to the ground. Darwin relates the case of a student, who, one day, returned home with haggard face and distracted look, and assured his companions that he would die in 36 hours. He took to his bed, made his will, and composed himself for death. Hufeland was called in. He ordered a dose of opium, under the influence of which the young man slept for more than the 36 hours. On awaking he was convinced of his hallucination, and owned that on going out on the day when he was attacked he had seen a death's head, and heard a voice say to him—" Thou wilt die in thirty-six hours."

A third patient requests us to remove the unpleasant smells which annoy him, or he is delighted with their exquisite fragrance. Yet there are no odours, nay the patient lost his sense of smelling prior to his illness. Or he is obliged to bite arsenic, to eat earth—flames scorch his mouth, or he quaffs ambrosia, or tastes the many viands of an exquisite repast.

Some feel points, or arms, perhaps, wounding them. They think that they have been beaten, nay, shew the imaginary bruises on their bodies.

Thus these patients believe in the presence of persons or of things which either do not exist, or are absent. Such persons are in a state of " hallucination."

M. Esquirol draws a distinction between hallucinations and the false perceptions of delirium. In the latter case an erroneous perception results from the impression of some object on the sense. A windmill, for example, is mistaken for a man. But in hallucinations, no real object impresses the sense at all. The idea originates within. Hallucination is therefore a mental act, accomplished independently of any present operation of the senses. There are many famous instances of men having displayed hallucinations, who evinced no other symptom of intellectual aberration.

The subject of hallucinations, like the dreamer, is so occupied with their consideration, that he cannot divert his attention from them. The habit of associating sensations with the objects that give rise to them, and this absorbing contemplation of the dominant hallucination, both conspire to convince the patient of its reality. He no more doubts it than we doubt a dream, while it endures. Hallucinations too, like dreams, as they draw on the memory of past impressions, always reproduce them. The series of images and ideas is occasionally regular; more frequently it is a strange association of them. Sometimes the individual is conscious that it is an hallucination which engages him, and yet he cannot shake it off.

Those who labour under hallucinations differ, for the most part, from somnambulists in this respect—that the latter remember nothing when the fit is over—while the former recollect all that they have undergone.

Hallucinations differ from ecstacy. The latter is always occasioned by some great effort of attention, in which the mind has been fixedly bent on some particular object or train of ideas. In ecstacy, so intense is the concentration of the nervous energy, that it absorbs all the powers of life, and all but imagination is suspended. It is otherwise with hallucinations. An augmented action of the nervous centre, rather than a violent effort of attention is requisite; and all the functions are performed with more or less regularity. The patient lives in the midst of his hallucinations as he would have lived in the midst of realities. The hallucination influences the thoughts, opinions, actions of the patient, who reasons on it as a truth.

When witches were burnt, many chose the stake rather than deny their guilt. It requires but a slight acquaintance with the history of witchcraft to know, that numerous unhappy wretches believed that they possessed the powers ascribed to them. M. Esquirol has known many persons who had been affected with hallucinations, remain, after their recovery, convinced of the reality of what they had seen or heard. "I saw and heard it all, as plainly as I see and hear you now," said a quondam *halluciné* to M. Esquirol. The hallucination affects him just as a corresponding truth would—he is convulsed with laughter—elated with joy—or sunk in hopeless melancholy.

The hallucination may present a constant character, or may range over the objects and ideas which memory calls up. When this is the case, the actions of the individual bear a corresponding impress of versatility. The same thing happens in some instances of mania, as well as in febrile delirium.

In reference to the seat of hallucinations, M. Esquirol places them, naturally enough, in the nervous centre—in some disturbance, primary or secondary, of the brain.

Hallucinations usually relate to the corporeal or intellectual occupations of the patient: or they display an evident connection with their cause. Thus, a female read the histories of witches, until she believed that she officiated at their sabbath, and witnessed all their orgies. A lady read the execution of a criminal—every where she saw a bloody head, wrapped in black crape. It always appeared above her left eye, filled her with unutterable horror, and drove her to several attempts at suicide.

Hallucinations may be the consequence of habitual exertions of the brain upon any particular topics. The mind accustomed to a certain train of thought, habitually falls into it to the exclusion of the present impressions of the senses, and this may go at last to the extent of actual hallucination. The *halluciné* presents the same *absence*, which characterises the meditative man of the soundest mind.

Love and religion being the sentiments that actuate the human breast most forcibly, the hallucinations of erotic and religious monomania are naturally the most frequent and the most remarkable.

Hallucinations occur in persons who have never been deranged; but they are also one of the elements of derangement, most frequently observed in mania, melancholia, ecstacy, catalepsy, hysteria, and febrile delirium. Out of a hundred insane, eighty, at least, have hallucinations.

Sometimes this symptom exists long before insanity is evident. Patients frequently struggle against it, while their conversation and their actions yet present no obvious deviation from propriety. Occasionally, the hallucinations are transitory and confused at the commencement, and only assume at a subsequent period a character of fixity and distinctness. Not uncommonly they remain after the derangement has ceased. In derangement of the most general character, the patient will all at once, perhaps, in the midst of an animated conversation, stop and fix his eyes on the imaginary object, or reply to the ideal persons whom he hears. This may be observed, more or less, in almost all cases of derangement. But those individuals are particularly prone to it, who, before their illness, were influenced by some predominant passion, or subjected to great mental disturbance, especially if addicted to abstruse and speculative inquiries. M. Esquirol, indeed, admits that hallucinations are most commonly observed in persons of weak minds; but those of the greatest vigour are by no means exempted from them.

The hallucination may be confined, as has been already hinted, to one sense, or it may extend to two, to all. The *halluciné* has been often looked upon as one inspired. In Germany he is still regarded as a seer. Many of the pretended prophets of the East, and we may add many of our own day in the West, are but the subjects of hallucinations. Subtracting the cheats, these are the remainder.

Hallucinations arising in impressions produced on the sense of taste or smell, are more particularly observed at the commencement of madness. Those connected with sight and hearing are common to all periods of it. The hallucinations of sight have popularly received the designation of visions. The term cannot be applied to any but visual hallucinations; yet those of smell, hearing, taste, have all the same generic character, own the same causes, have the same cerebral seat, occur in the course of the same diseases. It is on this account that M. Esquirol proposes the generic term *hallucination* as their representative.

Hallucinations being only a symptom of intellectual derangement, and common to many forms of it, require no special treatment. But their precise character must necessarily tinge the features of the disorder, and the moral and intellectual treatment which it requires.

### On the Illusions of the Insane.

Such is the title of the next chapter, or essay, of our author.

Illusions differ from hallucinations in this—that the former are false perceptions of real impressions on the senses—the latter, as our readers already know, being unconnected with present impressions on the senses altogether.

Certain illusions of sense are compatible with sanity of mind. Thus, a square tower seen from a great distance seems a round one; but then the error is dissipated by the diminution of distance.

Hypochondriacs have distorted perceptions of internal sensations. The intensity of their sufferings, their dangerous condition, are illusions. Yet hypochondriacs rather exaggerate impressions, than distort them in such a manner as to be inconsistent with reason. Decided melancholia indeed may be grafted on hypochondriasis, and then, of course, there is actual derangement of ideas. Hypochondriasis is, after all, but a minor degree of melancholia, and it is difficult to draw a definitive line between them.

Accurate perception implies three conditions—integrity of the organ of sense—integrity of the nerve which transmits the impression to the sensorium—and, finally, integrity of the sensorium itself. Illusions, which are false perceptions, may result from the disturbance of each of these conditions—from disorder of the organ of sense—of the nerve—of the encephalon.

If, in mania, the attention is insufficiently fixed on objects, and the mind wanders rapidly from point to point, the impression which they make is of necessity imperfect, and illusion the result. In monomania, the same effect results from a precisely opposite cause. There the attention is concentrated on a single object, its properties are magnified, comparison with other objects does not come to rectify the error, and exaggeration and caricature are the consequences.

The passions lend a liberal aid in the creation of illusory ideas. The source of so many illusions of the sane, how can they fail to modify the sensations of the insane? In point of fact, they are the parents of a thousand false perceptions in the latter.

As illusions may result from impressions felt within, or received from without, M. Esquirol divides them into ganglionic and sensible—those arising through the medium of the gangliar system, or of the organs of sense.

M. Esquirol relates several cases in illustration of each, but, with the utmost deference to his authority, they appear to us to be rather instances of the contrary of what he cites them for. As an example of illusions from internal sensations, he brings forward the case of extreme sensibility of the skin which is dry, harsh, burning, and incapable of properly performing its functions. An insane person, with such a state of skin, imagines that a touch is a blow, or a burn, that those around him are endeavouring to wound or torture him. But surely this is an illusion founded on deranged action or an altered condition of an organ of *sense*—the skin. The same objection lies against most of M. Esquirol's other cases, and however valuable the work of this gentleman may be, it displays, in common with those of most continental writers, a singular inconsequentiality of reasoning, and a strange mixture of accuracy and laxity of observation. We have often been struck, on perusing foreign works, with this circumstance. In a train of ratiocination, or of observations, the premises are arranged in the most methodical form, the necessary conclusions seem on the very point of appearing, when, presto, comes some extraordinary inference, totally at variance with all that went before, and diametrically opposed to the very train of reasoning it is placed amongst.

M. Esquirol relates several cases in which pain in various parts of the body gave birth to different illusions. We will notice one or two.

*Case.* Mademoiselle ————, aged 18, had enjoyed pretty good health, although her menses were irregular, when in 1815 she began to suffer from a fixed pain in the vertex. She soon persuaded herself that there was a worm devouring her brain. The sight of copper almost made her faint, and her relatives were obliged to remove every gilt ornament from her apartments. She was with difficulty induced to walk out, the dust raised by the passengers seeming to her charged with oxyde of copper. After several months of ineffectual treatment, M. Esquirol was consulted. She was attenuated, very irritable, sometimes refused to eat, slept badly, and laboured under constipation. She spoke of her aversions, sometimes with anger at other times with tears. M. Esquirol won her confidence by sympathising with her feelings, and persuaded her that he could cure her and had cured others by a trifling operation. The young lady was so excited, that after a visit from M. Esquirol she made an incision into her own scalp with a penknife. M. E. was summoned. The young lady requested the performance of the operation he had proposed. A crucial incision was made over the seat of pain, and a fragment of fibrine was shewn to the patient with the assurance that it was the worm. An issue was then established and kept open for three months. The illusion on the subject of the worm and the verdigris soon disappeared.

Another case was not so fortunate. A countrywoman was admitted into the Salpêtriere, complaining of acute pain at the vertex, which she attributed to the presence of an animal. She had fallen into melancholia, with a tendency to suicide. M. Esquirol made a crucial incision over the painful part, and shewed the patient a piece of an earth-worm, which he assured her had been the cause of all her sufferings. The patient, in her joy at being cured, shewed the worm to her companions. They laughed at her for her credulity, which made her so furious that she refused to submit to the establishment of an issue, her old pains returned, and the illusions were re-established with them.

M. Esquirol opened the body of a woman at the Salpêtriere, who had laboured under melancholia, and asserted that she had an animal in her stomach. There was cancer of that organ.

Another female, who, after an illicit amour with her master which was followed by an accouchement, experienced much dejection and frequent gastro-intestinal disorders, turned devotee. She ultimately became maniacal, and entered the Salpêtriere where she lived many years. She believed that she had Pontius Pilate in her belly, and occasionally he was joined there by all the personages of the New Testament, and indeed, of the Old. When the pains were more than usually acute, she quietly announced that she heard them nailing Jesus Christ to the cross. On examining her body, the abdominal viscera were found all matted together, and the peritoneum was greatly thickened.

Irritation, pains, or lesions in the generative organs, are the frequent sources of illusions in the insane, and more especially in women. They have led to mutilations of these organs. Females labouring under erotic monomania imagine themselves in the arms of their ravishers or lovers—or conceive that the devil, or that serpents are entering their bodies through the external organs. Cancer and ulcers of the uterus are not uncommon in these patients.

The vague pains that the insane experience in their limbs may lead to illusions of a very distressing character.

At the Charenton, there is a monomaniac, aged about thirty years, who believes that, every night, he is led to the cellars of the opera, where knives and daggers are plunged into his chest and back, his limbs amputated, and even his head cut off. If it is observed to him that this cannot be, for his head is still upon his shoulders,—"ah," he cries, "my persecutors are magnetisers, free-masons, who have the secret of fitting the amputated parts on again." Any further remonstrance only leads to violence. "You are leagued with them. Kill me, kill me. I cannot go through my cruel sufferings."

The illusions of the senses are striking. If the lunatic hears a noise, it is of voices addressing him—of friends flying to his rescue, or of enemies rushing to his destruction. M. Esquirol attended a lady whom the slightest sound threw into a paroxysm of terror. She trembled at the murmuring of the wind, and the noise which she herself made in bed alarmed her to such a degree, that she would rise and scream for assistance. She was got to sleep by having a light burning in her chamber and an attendant to watch over her.

The sight, more particularly, breeds these idle fancies. Nothing more likely, for sight is the great sense.

A lady, labouring under hysterical mania, was constantly at the window of her apartment. Whenever she saw an insulated cloud she cried to it, "Garnerin, Garnerin, come for me." She mistook the cloud for one of Garnerin's balloons.

An officer of cavalry believed that the clouds he saw, were an army headed by Napoleon for a descent upon Britain.

The insane often collect stones and bits of broken glass, which they think are diamonds, objects of vertù, or of natural history. They preserve them with the greatest care, and wonder, perhaps, at the ignorance of those who do not perceive their inestimable value.

The light and shade on the walls of the apartment may lead to singular illusions.

M—, labouring under melancholia, was in the habit of constantly striking at the furniture, &c. of the room in which he walked. The faster he walked, the harder he hit. At last M. Esquirol ascertained that the alternations of light and shade, occasioned by his transit through the room, created the illusion that there were rats in it. The faster he walked, the more, of course, the rats were multiplied.

M. Esquirol attended a young lady possessed of a lively imagination, who had devoted much attention to literature and to art. She had become maniacal, and passed her nights without sleep, in raptures at the beauty of the paintings which she saw upon the bed and window-curtains. On taking away the light, the illusion vanished.

M. Esquirol relates two other cases for the purpose of proving that the mere act of vision has much to do with similar illusions.

Reil relates the instance of a lady who had paroxysms of agitation, and even of fury. Her attendant, one day, in the endeavour to calm her, put her hands before her eyes. The patient immediately grew tranquil, and said that she saw nothing. Her physician repeated the experiment with the same result.

M. Esquirol attended a young soldier, allied to the family of Bonaparte. He had gone through many hardships, become maniacal, and was placed under M. Esquirol's care. He saw in all the persons around him members of the Imperial family, and was highly incensed at perceiving the domestics engaged in any servile occupation. He prostrated himself at the feet of one whom he took to be the Emperor, and claimed his pity and protection. M. Esquirol determined to try the effect of bandaging his eyes. The patient instantly grew calm, and could even discourse rationally on the subject of his illusions. M. Esquirol repeated this experiment on several occasions with the same result. On one, the bandage was kept on for twelve hours, during which there was no illusion. As soon as he was allowed to see, the latter re-appeared.

The sense of smell, gives rise to *its* lying brood. The insane often refuse their food, on account of the disagreeable odour in it; and they not unfrequently complain of noxious gases in the air.

M. Esquirol has seen lunatics when in a state of great disquiet or of agitation, soothed by the diffusion of agreeable odours in their rooms.

In almost all instances the commencement, and, in some, the course of mental diseases exhibit disturbance of the organs of digestion. These patients may find a bad taste in all the food presented to them. They suspect poison, and reject sustenance with horror or with rage. The suspicions which torment them make them conceive distrust and aversion of their friends or relatives, and greatly distress, of course, the latter. The symptom ceases after a few days, either under the influence of abstinence, or from the effect of a few evacuations. It need not occasion alarm, for in cause, in character, and gravity, it differs from the obstinate refusal to eat on the part of monomaniacs—a refusal originating in some dominant idea, such as an expiation, or a precept of religion or of honour, or a resolution to die.

The parched state of the mucous membrane of the tongue and mouth, give some lunatics the idea that earth is mingled with their food. In dementia, on the other hand, taste being destroyed, the patients eat with avidity the most filthy and disgusting substances.

When inflammation, or other lesion, has affected the meninges or the structure of the brain, the limbs of the insane sometimes tremble, while the extremities of the fingers lose their natural sensibility. Such patients are clumsy, seize things awkwardly, and do not hold them well. They break or let them fall, and judge erroneously of the form, extent, solidity, and weight of bodies.

A lady much debilitated by an accouchement and by sanguineous evacuations, resorted to for the purpose of combating an attack of mania, laboured under obstinate constipation. M. Esquirol prescribed lavements, which the lady insisted on giving to herself. Scarcely had she taken the syringe in her hand, when she threw it down with horror. This happened several times. The lady afterwards assured our author, that the syringe seemed to her so heavy, that she thought it filled with mercury, and was convinced that she was to be turned into a barometer.

M. Esquirol winds up this Essay by the following conclusions:—

1. Illusions are provoked by sensations originating within or without.

2. Illusions are the result of the action of the sentient extremities of the nerves, and of the re-action of the sensorium.

3. Illusions must not be confounded with hallucinations, in which the sensorium alone is at fault.

4. Illusions beguile the judgment on the nature and the cause of impressions actually made, and may drive the insane to acts fraught with danger to himself or to others.

5. Education, habit, profession, sex, modify the character of the illusion.

6. Illusions take their tone from the dominant passions and ideas of the insane.

7. Reason disperses the illusions which may occur in the man of sane mind, but it cannot dissipate those of the lunatic.

Such is the memoir of M. Esquirol on the subject of illusions. This and the preceding one, on hallucinations, are worth the attentive perusal of our readers.

